# A 10-year population-based study of people with multiple sclerosis in Stockholm, Sweden: use of and satisfaction with care and the value of different factors in predicting use of care

**DOI:** 10.1186/s12913-015-1144-1

**Published:** 2015-10-24

**Authors:** Charlotte Chruzander, Sverker Johansson, Kristina Gottberg, Ulrika Einarsson, Jan Hillert, Lotta Widén Holmqvist, Charlotte Ytterberg

**Affiliations:** Karolinska Institutet, Department of Neurobiology, Care Sciences and Society, Huddinge, Sweden; Karolinska Institutet, Department of Clinical Neuroscience, Stockholm, Sweden; Department of Physiotherapy, Karolinska University Hospital, Stockholm, Sweden; Department of Neurology, R54, Karolinska University Hospital, Huddinge, SE-141 86 Stockholm, Sweden

**Keywords:** Multiple sclerosis, Health care, Patient satisfaction, Longitudinal, Population-based, Utilization, Observational

## Abstract

**Background:**

The national strategy for treatment of chronic diseases - including MS - and changes in the Swedish welfare system, call for analyses of the use of, and patient satisfaction with, care in a long-term perspective. The aim was therefore to explore the use of care and the predictive value of personal factors, disease-specific factors and functioning on the use of care and to explore patient satisfaction with care in a 10-year perspective.

**Methods:**

Information regarding personal factors, disease-specific factors, functioning and satisfaction with care was collected by home-visits; use of care was collected from the Stockholm County Council computerised register.

**Result:**

Data from 121 people with MS (PwMS) was collected. Primary care accounted for the majority of all care. Neurology and Rehabilitation Departments together accounted for two-thirds of all hospital outpatient care. Rehabilitation Departments accounted for one-third of the total number of inpatient days. Lower coping capacity, impaired manual dexterity and activity of daily living dependency at baseline, together with progress in MS disability predicted a higher use of care. Overall, patient satisfaction with care was stable over time.

**Conclusion:**

The extensive use of care offers challenges to care coordination. Implementation of person-centred care could be a strategy to increase efficacy/outcome of care.

## Background

Multiple sclerosis (MS) is a neuroinflammatory and neurodegenerative disease, characterized by demyelination and axonal degeneration in focal areas of the central nervous system [1, 2]. MS is commonly diagnosed in people who are 20–40 years of age and is the leading cause of neurological disability in younger adults. The disease may cause a wide range of symptoms including fatigue, motor- and cognitive impairments, sensory disturbance, depressive symptoms, bladder, bowel and sexual impairments [3] with a significant impact on quality of life, working ability and ability to fulfill household responsibilities [4].

Since MS has a great impact on functioning in people with MS (PwMS), the majority will need health care over a period of many years. Previous cross-sectional and short-term (up to three years) studies have found that PwMS use a large amount of care [5, 6] and that a more severe MS is associated with a higher cost and a higher total amount of care used [5]. It has also been found that depressive symptoms [7] and fatigue [8] are associated with a higher use of care, but the impact of other variables has not been explored. Studies have demonstrated that PwMS are not satisfied with a number of areas of care, for example; accessibility of care [9] and psychosocial support [10, 11]; advice on social security matters; and continuity of rehabilitation services [10].

Within the Stockholm MS-study, a population-based study of PwMS in Stockholm, Sweden, baseline cross-sectional results showed a parallel use of care within many different departments and services and a large proportion of the PwMS were not satisfied with the supply of psychosocial support/counselling from the health care system [12].

In the light of the recent Swedish national strategy for prevention and treatment of chronic diseases - including MS - aiming to develop care for persons with chronic diseases [13], and the changes in the Swedish welfare system conducted in order to enhance productivity and efficiency and to be more sensitive to the patients’ preferences [14], we have performed analyses of mortality, disability and HRQL within a 10-year follow-up of the Stockholm MS-study. Results showed that 19 % of the PwMS had died and that older age, a progressive disease course and depressive symptoms were associated with mortality [15]. In those who survived, there was a significant increase in the proportion of PwMS with disability in walking speed, manual dexterity, and in the proportion of PwMS who were dependent in Activities of Daily Living (ADL) [15]. However, the Health Related Quality of Life (HRQL) was quite stable over time [16].

To be able to provide means for improvement it is important to analyse also the use of, and satisfaction with, care among PwMS in a long-term perspective. Thus, the aims of this 10-year follow-up of the Stockholm MS-study were to: a) explore the use of care and the predictive value of personal factors, disease-specific factors and functioning on the use of care, and b) explore the satisfaction with care from the perspective of the PwMS.

## Methods

### Participants

The present study is a 10-year follow-up of the Stockholm MS-study for which the case-finding procedure has been described previously [17]. In brief, those PwMS included at baseline (from September 1999 to September 2002) were recruited from a temporary data pool comprising of 2129 patients from all neurological hospital clinics in Stockholm County, in order to obtain the utmost possible population-based ascertainment. A random sample representing 15 % (*n* = 321) of the data pool was drawn. Inclusion criteria were a definite and informed MS diagnosis, residence in Stockholm County and no diagnosis of other severe neurological or psychiatric illness. In total 196 PwMS fulfilled the criteria and 166 (85 %) of them gave informed consent, both verbally and in written form, to participate in the baseline study. For the purpose of data collection in the 10-year follow-up, the same PwMS were identified and those still alive were contacted through a postal letter.

### Data collection procedures

The data collection procedures at baseline, regarding personal and disease specific characteristics, functioning [17] and satisfaction with care [12] have been described previously as have the data collection procedures at the 10-year follow-up [15]. In brief, data collection was performed at baseline (from 1999 to 2002) and 10 years ± 6 months after using structured face-to-face interviews during home visits. Each home-visit lasted for two to three hours and was conducted by health care professionals, all with clinical experience in neurologic assessment and calibrated for the purpose of data collection.

Table 1 presents the independent variables used in the univariable and multivariable linear regression analyses, methods and instruments used for data collection and criteria for categorisation of the variables. The following variables were collected at baseline: coping capacity; level of education; MS disability; disease course; time since diagnosis; use of immunomodulatory treatment; mood; cognitive function; manual dexterity; walking ability; capacity in personal and instrumental ADL and frequency of social/lifestyle activities. In addition, progress in MS disability was defined as more than one point difference from baseline to the 10-year follow-up according to the Expanded Disability Status Scale (EDSS) [18].

**Table 1:** Independent variables used in the linear regression analyses, variables and instruments used for data collection, and categorisation of the variables

Variables and instruments	Categorisation
Age	Continuous
Sex	Female/Male
Coping capacity:	
Sense of Coherence Scale [30]	Continuous or
	Weak: 13–54 points/Moderate to Strong: 55–91 points [31]
Education:	
Interview	Primary or lower secondary level/High School or University
Degree of MS disability^a^:	
Expanded Disability Status Scale [18]	Mild: 0–3.5/Moderate: 4.0–5.5/Severe: 6.0–9.5
Type of MS^b^	Relapsing/remitting MS/Progressive MS
Time since MS diagnosis^c^	Continuous
Immunomodulatory treatment^c^	Yes/No
Mood:	
Beck Depression Inventory II [32]	No depressive symptoms: <13/Depressive symptoms: ≥13 [28]
Cognitive function:	
Symbol Digit Modalities Test [33]^d^	Age-related norms, written or oral reply. No impairment: < −1.5 SD from the mean/Impairment: ≥ −1.5 SD from the mean [33]
Manual dexterity:	
Nine Hole Peg Test [34]^e^	No impairment: ≥0.5 pegs/sec/Impairment: <0.5 pegs/sec [33]
Walking ability	Walk without aid/Walk with aid/Cannot walk
Personal activities of daily living:	
Barthel Index [35]	Independent: 100 points/Dependent: < 100 points
Personal and instrumental activities of daily living:	
Katz Extended ADL Index [36]	Independent: 20 points/Dependent: < 20 points
Frequency of social/lifestyle activities:	
Frenchay activities index [37]	Age-, sex-related norms. No impairment: >25^th^ percentile/Impairment: < 25^th^ percentile [38]
Progress in MS disability:	
Expanded Disability Status Scale	No change: ≤1 points change/Change: >1 points change

^a^Assessed by the data collectors and verified by a senior neurologist

^b^Progressive MS includes those with either primary progressive MS or secondary progressive MS

^c^Data was collected through medical records

^d^Primarily conducted with written response, for those PwMS unable to write the test was administrated orally

^e^For the right hand

Data on the use of primary care, hospital outpatient care and hospital inpatient care were obtained from the computerised register at Stockholm County Council. The register contains information regarding all care use (clinical visits and home visits, telephone consultations and inpatient days) with care providers within Stockholm County Council.

To assess satisfaction with care, a questionnaire used in previous studies [12, 19, 20] and based on the taxonomy of Ware was used [21]. The questionnaire includes the following dimensions: art of care; technical quality of care; accessibility/convenience; finances; availability; continuity and efficacy/outcomes of care. In addition, items relating to participation in the planning of care were included in the questionnaire. The questionnaire consists of 18 items constructed as statements, with which the PwMS agrees or disagrees (satisfied-dissatisfied) on a five-graded Likert scale. After the PwMS had filled in the questionnaire, the answers were dichotomised into “not satisfied” (1–3 on the Likert scale) or “satisfied” (4–5 on the Likert scale).

### Statistics

Descriptive statistics were used to analyse use of care (primary care; hospital outpatient care; inpatient care) distributed by profession or department from baseline to the 10-year follow-up.

Univariable and multivariable linear regression analyses with a stepwise backward selection were performed in order to analyse the predictive value of the independent variables at baseline and progress in MS disability on the use of care. Data on the use of primary care and hospital outpatient care (clinical visits, home visits, telephone consultations) were summarised into the dependent variable “total outpatient care” and data on all inpatient care (days) formed the dependent variable “inpatient care”. Because of highly skewed distribution of the dependent variables, the data was log transformed. One PwMS had only one contact in total outpatient care and was therefore excluded from the regression analyses of total outpatient care. For each dependent variable a stepwise analysis including all independent variables was performed. To obtain the models with the best fit, separate multivariable analyses were performed using the independent variables age, coping capacity and time since diagnosis as either continuous or categorised. The models with the highest adjusted coefficient of determination were then selected. For total outpatient care, coping capacity was used as a continuous variable and for inpatient care, coping capacity was used as a categorised variable. The criterion for removing variables was set to a *p* value > 0.1. The final results of the multivariable linear regression analyses are presented as regression coefficient (B) with a 95 % confidence interval (CI), standardised regression coefficient (Beta) and *p* value.

Changes in satisfaction with care in those PwMS who reported a need at both baseline and at the 10 year follow-up were analysed using the McNemar test. If fewer than five PwMS had expressed a need at both baseline and at the 10-year follow-up, no statistical analysis was performed for that item. The *p* value for a change was set to ≤ 0.05. To explore the use of care for PwMS satisfied versus PwMS not satisfied with the efficacy/outcome of primary care, hospital outpatient care, inpatient care and inpatient rehabilitation, the Mann Whitney *U* test was used. The *p* value for a difference was set to ≤ 0.05.

The Regional Ethical Review Board in Stockholm approved the study, dnr: 2009/3:3.

## Results

A total of 166 PwMS were included at baseline, of which 32 were deceased at the 10-year follow-up. An additional 12 PwMS declined to participate and one PwMS had moved from Stockholm County and was therefore excluded. Consequently, 121 PwMS were included in the present study. Of these 121 PwMS, four were unable to be interviewed in the 10-year follow-up due to severe disability and were therefore not included in the analysis of satisfaction with care over time. Personal- and disease-specific characteristics, proportions of PwMS with disability at baseline, and progress in MS disability are presented in Table 2.

**Table 2:** Personal and disease-specific characteristics in the sample and number and proportions of people with MS with disability at baseline, and number and proportions of people with MS with progress in MS disability (*n* = 121)

Variable	n (%)
Mean age^a^	49 (11)
Female	83 (69)
Upper secondary school or university level	89 (74)
Comorbidity	45 (37)
Degree of MS disability^b^	
Mild	41 (34)
Moderate	24 (20)
Severe	56 (46)
Type of MS	
Relapsing/remitting	55 (45)
Progressive	66 (55)
Time since MS diagnosis^a^	18 (11)
Immunomodulatory treatment	48 (40)
Depressive symptoms	21 (19)
Cognitive impairment	49 (47)
Impaired manual dexterity	64 (54)
Walking ability	
Walk without aid	75 (64)
Walk with aid	22 (18)
Cannot walk	21 (18)
Dependent in personal activities of daily living	47 (40)
Dependent in instrumental activities of daily living	73 (61)
Reduced frequency of social/lifestyle activities	66 (56)
Progress in MS disability	58 (48)

^a^Years (sd)

^b^According to the Expanded Disability Status Scale

**Fig. 1:**
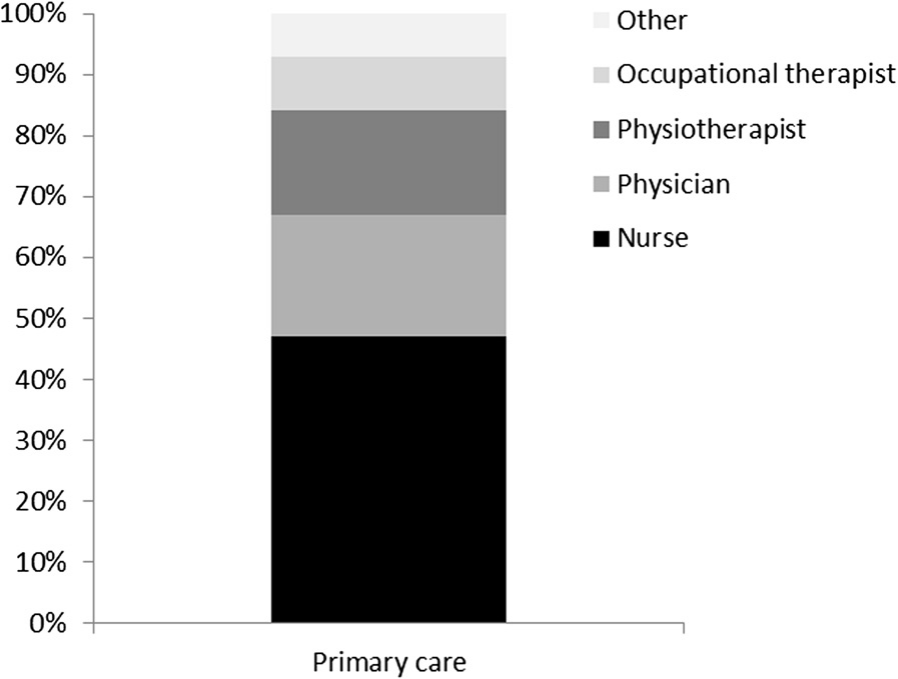
Proportion of contacts with primary care (*n* = 23 379 contacts), in people with MS during the 10-year period, distributed by profession. Other includes professions with less than 5 % of the total number of contacts (e.g. unspecified, biomedical analysts, audiologist, speech therapist, psychologist, podiatrist, dietician, welfare officer, nursing assistance, orthoptist)

All PwMS had been in contact with primary care. Nurses composed the largest proportion of all use in primary care (Fig. 1). Almost all PwMS had been in contact with physicians and nurses and fewer with occupational therapists and physiotherapists during the 10-year study period (Table 3).

**Table 3:** Use of primary care in people with MS during the 10-year period distributed by health care profession: number and proportion of users (%), mean (sd), median (IQR) and range of contacts (clinical visits, home visits, telephone consultations)

	Users, n (%)	Mean (sd)	Median (IQR)	Range
Total use of primary care	121 (100)	192 (297)	91 (33–210)	1-1884
*Profession*				
Physician	117 (97)	39 (52)	26 (10–43)	1–299
Nurse	113 (93)	91 (213)	20 (5–93)	1–1428
Occupational therapist	73 (60)	30 (30)	21 (7–43)	1–140
Physiotherapist	61 (53)	62 (117)	17 (3–85)	1–789
Nurse aid	24 (20)	37 (139)	4 (1–15)	1–689
Orthoptist	15 (12)	12 (11)	11 (2–18)	1–43
Welfare officer	12 (10)	6 (5)	4 (2–11)	1–16
Dietician	10 (8)	4 (4)	2 (1–7)	1–14
Psychologist	7 (6)	10 (7)	10 (4–14)	2–23
Podiatrist	7 (6)	16 (13)	12 (6–27)	1–40
Other^a^	15 (12)	6 (8)	2 (2–9)	2–25

^a^Includes professions that less than 5 % of the people with MS have been in contact with

**Fig. 2:**
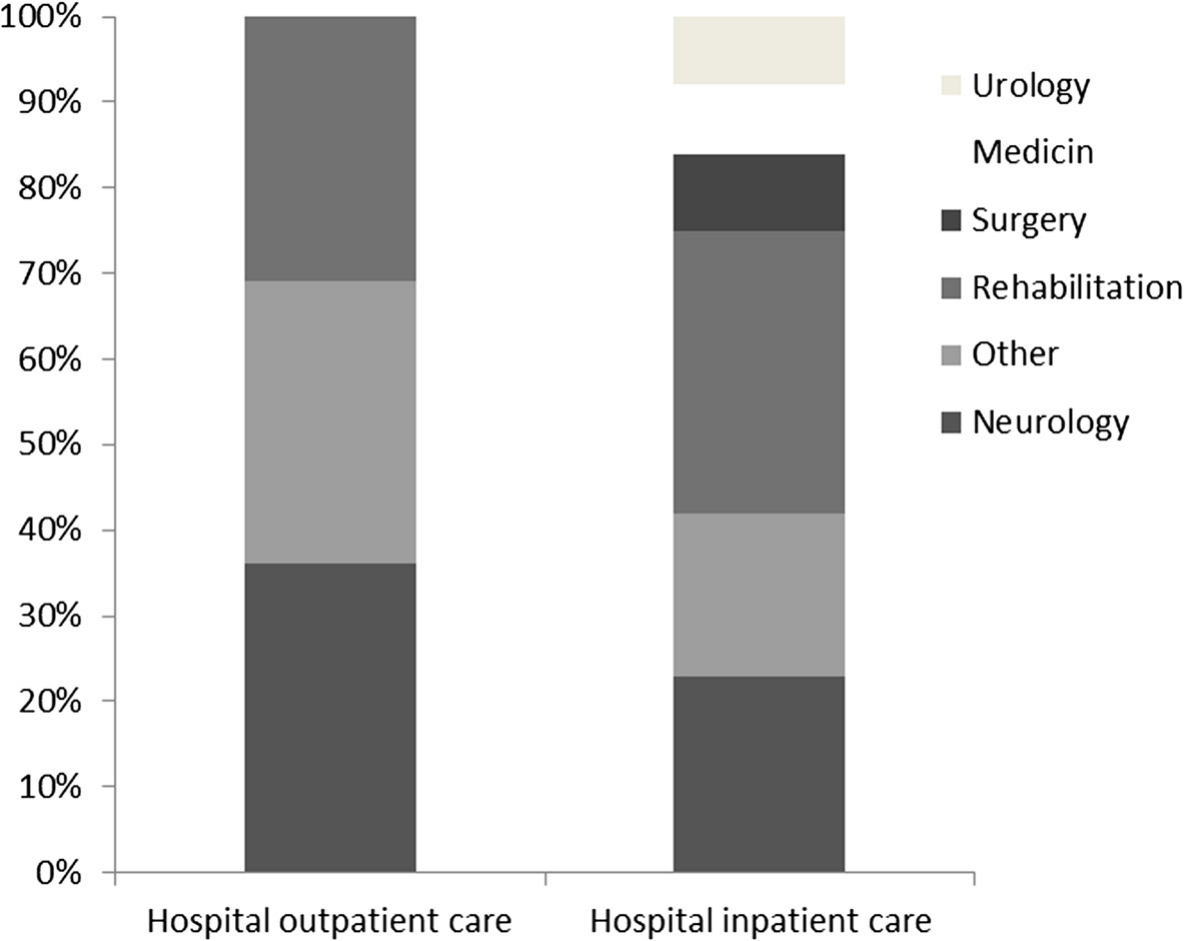
Proportion of contacts with hospital outpatient care (*n* = 12 706 contacts) and hospital inpatient care (*n* = 12 706 days), in people with MS during the 10-year period, distributed by department. Other includes departments with less than 5 % of the total number of contacts/days (e.g. orthopedics, hematology, infection, gastroenterology, psychiatry, geriatrics, ear, nose and throat, emergency room and advanced home care)

Neurology Departments and Rehabilitation Departments composed two thirds of all use in hospital outpatient care (Fig. 2). Almost all PwMS had been in contact with Neurology Departments (Table 4).

**Table 4:** Use of hospital outpatient care in people with MS during the 10-year period distributed by department: number and proportion of users, mean (sd), median (IQR) and range of contacts

Department	Users, n (%)	Mean (sd)	Median (IQR)	Range
Total	120 (99)	106 (86)	72 (47–149)	9–543
Neurology	117 (97)	39 (31)	30 (16–50)	1–160
Medical service and radiology	103 (85)	5 (4)	4 (2–6)	1–23
Rehabilitation	92 (76)	42 (66)	18 (5–42)	1–350
Emergency	83 (69)	5 (6)	3 (1–7)	1–34
Urology	67 (55)	13 (16)	7 (4–17)	2–59
Ophthalmology	65 (54)	4 (4)	3 (1–6)	1–15
Obstetrics and gynecology	63 (52)	5 (5)	4 (1–6)	1–23
Surgery	61 (50)	7 (14)	3 (1–6)	1–79
Medicine	56 (46)	5 (7)	3 (1–6)	1–40
Orthopedics	50 (41)	4 (5)	2 (1–4)	1–30
Ear, nose and throat	30 (25)	2 (2)	2 (1–3)	1–9
Dermatology	21 (17)	2 (1)	1 (1–3)	1–4
Oncology	19 (16)	13 (17)	5 (2–23)	1–56
Geriatrics	17 (14)	5 (10)	2 (1–4)	1–41
Infection	13 (11)	5 (8)	1 (1–6)	1–30
Gastroenterology	11 (9)	7 (12)	2 (1–9)	1–33
Psychiatry	10 (8)	11 (19)	2 (1–31)	1–40
Endocrinology	8 (7)	3 (3)	2 (1–3)	1–10
Other^a^	56 (46)	14 (17)	7 (2–22)	1–71

^a^Includes professions that less than 5 % of the PwMS have been in contact with

Rehabilitation Departments composed one third of all inpatient care, followed by Neurology Departments which composed almost one third of all inpatient care (Fig. 2). Three quarters of all PwMS had been hospitalised at some point during the study period (Table 5).

**Table 5:** Use of hospital inpatient care in people with MS during the 10-year period distributed by department: number and proportion of users, mean (sd), median (IQR) and range of inpatient days

Department	Users, n (%)	Mean (sd)	Median (IQR)	Range
Total	93 (77)	30 (47)	12 (4–37)	1–302
Neurology	39 (32)	23 (33)	12 (5–25)	1–167
Rehabilitation	38 (31)	38 (41)	26 (20–39)	2–222
Medicine	31 (26)	11 (12)	6 (3–16)	1–49
Surgery	30 (25)	12 (23)	5 (3–14)	1–132
Urology	16 (13)	21 (32)	6 (2–33)	1–120
Orthopedic	15 (12)	7 (8)	5 (2–8)	1–35
Infection	13 (11)	13 (19)	7 (4–15)	1–71
Geriatrics	10 (8)	17 (10)	14 (10–26)	6–37
Obstetrics and gynecology	6 (5)	7 (6)	6 (4–10)	1–18
Other inpatient care^a^	11 (9)	42 (82)	7 (4–98)	2–189

^a^Includes departments which less than 5 % of the people with MS had been in contact with

A total number of 35,970 contacts were registered for primary care and hospital outpatient care from baseline to the 10-year follow-up. Sixty-five percent of these contacts were registered in primary care and 35 % were registered in hospital outpatient care. The final multivariable regression analysis for the use of total outpatient care revealed that a lower coping capacity, use of immunomodulatory treatment, impaired manual dexterity, inability to walk, and dependence in instrumental ADL at baseline together with a progress in MS disability predicted a higher use of total hospital outpatient care (Table 6). The adjusted coefficient of determination for the final multivariable linear regression model was 0.340.

**Table 6:** The final multivariable linear regression model for the predictive value of the independent variables at baseline, and progress in MS disability, on the use of total outpatient care during the 10-year period in people with MS (*n* = 120^a^)

Independent variable	Categorisation of the independent variable	B	95 % CI	Beta	*p* value
Coping capacity	Continuous	−1.30	−2.74 to 0.20	0.15	0.08
Immunomodulatory treatment	No	−41.76	−98.38 to −1.31	0.17	0.04
	Yes	reference			
Manual dexterity	Impaired	43.33	−4.29 to 114.26	0.18	0.08
	Not impaired	reference			
Walking	Cannot walk	105.01	23.86 to 239.06	0.26	0.01
	Walk without aid	reference			
Instrumental activities of daily living	Dependent	108.55	36.34 to 218.67	0.36	0.001
	Independent	reference			
Progress in MS disability	>1 point change	48.59	5.76 to 108.76	0.20	0.02
	≤1 point change	reference			

Adjusted coefficient of determination = 0.340

^a^One person had no contacts with hospital outpatient care and was excluded from the multivariable regression analysis

The final multivariable regression analysis for the use of inpatient care revealed that a weak coping capacity, impaired manual dexterity and dependence in personal ADL at baseline together with a progress in MS disability predicted a higher use of inpatient care (Table 7). The adjusted coefficient of determination for the final multivariable model was 0.280.

**Table 7:** The final multivariable linear regression model for the predictive value of the independent variables at baseline, and progress in MS disability, on the use of hospital inpatient care during the 10-year period in people with MS (*n* = 121)

Independent variable	Categorisation of the independent variable	B	95 % CI	Beta	*p* value
Coping capacity	Weak	167.51	12.64 to 535.18	0.19	0.03
	Moderate/strong	reference			
Manual dexterity	Impaired	233.01	82.58 to 507.30	0.36	<0.001
	Not impaired	reference			
Personal activities of daily living	Dependent	126.60	20.80 to 324.61	0.24	0.01
	Independent	reference			
Progress in MS disability	>1 point change	145.22	39.38 to 331.89	0.27	0.002
	≤1 point change	reference			

Adjusted coefficient of determination = 0.280

Overall, the proportion of PwMS satisfied with different dimensions of care was similar at baseline and at the 10-year follow-up although the proportion of PwMS who were not satisfied with the accessibility to rehabilitation periods; the accessibility to psychosocial support and advice/support of social insurance/work rehabilitation; the availability of physicians; and the proportion who had participated in planning care ranged between 34 and 66 % at both baseline and at the follow-up. However, there was a significant increase in the proportion of PwMS satisfied with: the accessibility of rehabilitation periods and home help service/personal assistance; the availability of nurses; and the efficacy of hospital outpatient care. No decrease in satisfaction with care over time was seen (Table 8).

**Table 8:** Proportion of people with MS satisfied with health care at baseline and at the 10-year follow-up, *p* value for differences of proportion satisfied at baseline and at the 10-year follow-up (*n* = 121)

Dimensions and related matters	Baseline, %	10-year follow-up, %	*p* value*
*Art of care*			
Sympathy/engagement from staff			
Physicians	67	72	0.62
Nurses	86	84	1.00
Physiotherapists	82	81	1.00
Occupational therapists	74	77	1.00
Welfare officers	76	78	0.29
Psychologists	67	87	
Others	84	75	
Kind treatment			
Physicians	89	92	1.00
Nurses	94	91	0.69
Physiotherapists	89	90	1.00
Occupational therapists	88	89	0.29
Welfare officers	89	90	1.00
Psychologists	81	88	
Others	86	88	
*Technical quality of care*			
*Accessibility*			
Physiotherapy	70	73	1.00
Occupational therapy	63	76	0.62
Rehabilitation periods	42	66	0.02
Assistive devices	89	90	1.00
Workplace adaption	59	67	0.27
Health-related transportation services	94	95	
Home adaptions	80	88	0.18
Home help service or personal assistance	63	85	0.01
Psychosocial support/counselling	47	53	0.69
Advice/support of social insurance/work rehabilitation	24	43	0.23
*Availability*			
Physicians	42	45	0.29
Nurses	69	80	0.04
Physiotherapists	81	85	1.00
Occupational therapists	64	80	1.00
Welfare officers	73	89	
Psychologists	62	91	
Others	69	86	
Contact with all expertise needed	76	76	0.87
*Continuity*			
Meeting the same staff	74	79	0.60
*Finances*	62	70	0.36
*Efficacy/outcome of care*			
Hospital inpatient care	76	88	0.61
Hospital out-patient care	75	85	0.05
Primary care	71	71	0.58
Rehabilitation	85	86	1.00
*Participation in planning care*			
Want to participate in planning care	90	86	0.33
Have participated in planning care	51	61	0.41

*If fewer than five PwMS had expressed a need at both baseline and at the 10-year follow-up, no statistical analysis was performed for that item

There was no difference in the use of care among PwMS satisfied compared to PwMS not satisfied with the efficacy/outcome of primary care (*p* = 0.08), hospital outpatient care (*p* = 0.99), hospital inpatient care (*p* = 0.49) and inpatient rehabilitation (*p* = 0.40).

## Discussion

This study revealed that, over a period of 10 years, all PwMS had been in contact with primary care which also accounted for the majority of all care used. Almost all PwMS had been in contact with hospital outpatient care and the Neurology Departments and the Rehabilitation Departments accounted for two-thirds of all hospital outpatient care used. Three-quarters of the PwMS had used hospital inpatient care at some point during the study period and the Rehabilitation Departments accounted for one-third of the total number of inpatient days. The results of the multivariable regression analyses for the use of total outpatient care and inpatient care respectively were similar with a lower coping capacity, disability in manual dexterity and dependence in ADL at baseline, and progress in MS disability as predictors for a higher use. Overall, the proportion of PwMS satisfied with care was stable over time, however, the high proportion of PwMS not satisfied with the accessibility to and the availability of certain types of care as well as the high proportion of PwMS who had not participated in planning their care indicate room for improvement. The use of care was not associated with satisfaction with the efficacy/outcome of care.

The great proportion of primary care used, especially nurses, could reflect the onset of secondary complications and a need for nursing care in the later stages of the disease. However, considering the great proportion of PwMS with comorbidity the high use of primary care is probably not solely MS-specific.

Even though MS is a neurologic and progressive disease, the Neurology Departments did not account for more than about one-third of all hospital outpatient care. Compared to other European countries the number of neurologist per thousand inhabitants in Sweden is low [22] which could explain the high proportion of PwMS not satisfied with the availability of physicians.

People with chronic diseases have been found to use more emergency care than people without a chronic disease [23] although this was not found in this study where only 4 % of the PwMS had visited the Emergency Room over 20 times or more during the 10-year study period. Rehabilitation accounted for a high proportion of the care used and even though there was a significant increase in the proportion of PwMS satisfied with the accessibility to rehabilitation periods from baseline to the 10-year follow-up, about one-third of the PwMS were still not satisfied with the accessibility to rehabilitation periods. Forty % of the PwMS in this study also experienced that they had not participated in the planning of their care. In Stockholm County, the Neurology Departments have the main responsibility for MS specific care while primary care has the overall responsibility for the total care of the PwMS. The extensive use of care during the 10-year study period highlights the challenge for primary care to coordinate all care between primary care and hospital outpatient care. Strategies to enhance coordination between caregivers are therefore warranted and need to incorporate the PwMS as full partners to their care-providers with a role in health care decisions [24] i.e. to establish a patient-centred care. In addition, new digital technology, such as the web-based platform linked to the Swedish MS-register, may enhance patient participation and patient-centeredness by enabling patients, including PwMS, to communicate with care providers.

Compared to another study exploring the use of care in PwMS in Sweden [25], our study reported a lower use of total outpatient care and number of inpatient days. In an international perspective there are studies reporting both a lower and a higher amount of visits to neurologists [26, 27] compared to our study, and a higher use of inpatient days at the Neurology Departments [27]. Differences in results might be attributable to differences in study sample, types of data collection, and length of follow-up. These differences as well as differences in organisation of care between countries challenge comparisons of results.

The prediction models of the use of care explained about one-third of the variance of the use of outpatient care and one-quarter of the use of inpatient care. Even though other variables not included in the models contribute to the variance of the use of care, the similarity between the models strengthens our results. Previous reports in Sweden have highlighted that age, sex and level of education [23] are important variables for the use of care, although this was not found in our study.

Even though the high prevalence of depressive symptoms in PwMS is well-known [28] and there are an increasing number of studies exploring the effectiveness of different psychological treatment methods of depressive symptoms in PwMS [29], the satisfaction with the accessibility to psychosocial support/counselling was low. In addition, the majority of the PwMS with a perceived need were not satisfied with the accessibility to advice/support of social insurance/work rehabilitation. This indicates the urgent need to gain knowledge about sickness absence and disability pension over the MS trajectory as well as to develop interventions aiming retaining PwMS in the work force.

This study has several strengths including the population-based cohort, the longitudinal design, and the possibility to link patient characteristics to all register-based outcomes. However, 19 % of the baseline cohort died during the 10-year follow-up time and these PwMS were not included in this study. It is possible that the use of care would have been higher if those PwMS who were deceased during the 10-year study period were included in the analyses. Furthermore, this study was conducted in Stockholm County, an urban area including a university hospital and with one of the highest number of neurologists per citizen in Sweden. It is possible that the distribution of care differs compared to more rural areas of Sweden and between Sweden and other countries.

## Conclusions

The result from this study is a significant knowledge base in order to create a long-term sustainable, effective and equal care for PwMS. The extensive use of care in different care sectors and in many different departments offers challenges to care coordination, and thereby also challenges the delivery of high quality care to the PwMS. By establishing the PwMS as full partners to their care providers in health care decision and implement strategies to coordinate care between primary care and outpatient hospital care there is a potential to increase efficacy/outcome of care.
